# Statistical corrections of linkage data suggest predominantly *cis *regulations of gene expression

**DOI:** 10.1186/1753-6561-1-s1-s145

**Published:** 2007-12-18

**Authors:** Jianxin Shi, David O Siegmund, Douglas F Levinson

**Affiliations:** 1 Department of Psychiatry and Behavioral Sciences, Stanford University, 701A Welch Road, Suite 3325, Stanford, California 94305, USA; 2 Department of Statistics, Stanford University, 390 Serra Mall, Sequoia Hall, Stanford, California 94305, USA

## Abstract

Morley et al. (*Nature *2004, **430:**743–747) detected significant linkages to the expression levels of 142 genes (of 3554) at a reported threshold of genome-wide *p *= 0.001 (LOD ≈ 5.3), using 14 three-generation Centre d'Etude du Polymorphisme Humain pedigrees. Most of the linkages (77%) were *trans*, i.e., more than 5 Mb from the expressed gene. However, the analysis did not account for the expected anti-conservative effect of the skewed distribution of score- or regression-based statistics in large sibships, or for the possible variance distortion due to correlations among tests. Therefore, we re-analyzed their data, using a robust score statistic for the entire pedigrees and correcting the *p*-values for skewness. We found that a LOD of 5.3 had a skewness-corrected genome-wide *p*-value of 0.016 instead of 0.001 (a result that we confirmed using simulation), with around 50 expected false positives. We then further corrected for correlation among the (skew-corrected) *p*-values by using Efron's method for obtaining the empirical null distribution. Setting a threshold of FDR = 10% (*Z *= 6.4, LOD = 8.9), we detected linkage for the expression levels of 22 genes, 19 of which are *cis*. Limiting the analysis to *cis *regions, linkage was detected to the expression levels of 46 genes with 4.6 expected false positives (FDR = 10%).

## Background

In their study of genome-wide linkage of expression levels of 3554 genes, Morley et al. [1] determined a genome-wide *p*-value for each phenotype using Gaussian process theory, and then used a form of Bonferroni correction, without accounting for dependencies among the many phenotypes being tested, to estimate the number of expected false positives among their 142 positive findings. However, Tang and Siegmund [2] have pointed out that in large sibships, because of the dependencies among identity-by-descent (IBD) counts, score- or regression-based statistics have a skewed distribution under the null hypothesis of no linkage, even if the phenotypes are exactly normally distributed. They have also provided a skewness-corrected approximation to the genome-wide *p*-value, which shows that approximations based on Gaussian processes can be quite anti-conservative in small samples. Also, Morley et al. [1] reported (and we have also observed, data not shown) that there are substantial correlations of expression levels for many pairs of genes in their data. Efron [3] has shown that correlation among many tests, if ignored, can lead to an excess or a deficit of significant findings, and he proposed a method to correct for this effect.

Therefore, we re-examined the linkage results of Morley et al. [1], using a robust score statistic to map the expression phenotypes, based on IBD counts for all relative pairs in each of the 14 Centre d'Etude du Polymorphisme Humain pedigrees. (Note that analyzing entire pedigrees is more powerful here than considering only the sibships; see below.) We corrected the *p*-values using the method of Tang and Siegmund [2] and then determined the false-discovery rate (FDR) using the method of Efron [4] to correct for the correlations among tests. We compared the results of this analysis to those based on a permutation-based FDR procedure. Finally, because most of our significant linkage signals were in *cis *regions (defined by Morley et al. [1] as within 5 Mb of the expressed gene), we also determined whether our analysis would have been more powerful if we had only tested linkage of each expression trait to the markers within 5 Mb of the gene.

## Methods

### A robust score statistic to map quantitative trait loci (QTL) using extended pedigrees

For notational simplicity, we suppress the index for each family. Let *Y *denote the phenotype for the members of a pedigree. Let *ν*_*ij*_(*t*) denote the number of alleles IBD at locus *t *between individual *i *and *j*, centered to have expected value 0. Let *A*_*ν*_(*t*) be the IBD matrix with [*A*_*ν*_(*t*)]_*ij *_= *ν*_*ij*_(*t*). Define Σ to be the phenotypic covariance matrix. Assuming no dominant genetic effect, then according to Tang and Siegmund [2], the conditional covariance matrix

Σ_*A *_= Cov(*Y*, *Y *| *A*_*ν *_(*τ*)) = Σ + *αA*_*α*_(*τ*),

where *α *≥ 0 denotes the additive genetic effect.

From the working assumption that at a trait locus *τ*, conditional on *A*_*ν*_(*τ*), *Y *follows a multivariate normal distribution, one can derive a robust score statistic for testing whether there is an additive genetic effect at *τ *[2] in the form *Z*(*τ*) = *l*_*α*_(*τ*)/[*E*_0_$l_{\alpha }^{2}$(*τ*)]^1/2^. Here,

*l*_*α*_(*τ*) = 2^-1^∑[-*tr*Σ^-1^*A*_*ν *_+ *tr*Σ^-1^*A*_*ν*_Σ^-1^*YY'*].

In practice, unobserved values of the IBDs in *l*_*α*_(*t*) are replaced by their conditional expectation given the genotypic data, while their variances are estimated from multipoint genotypic data. To make the test robust to the normality assumption of the traits, we use *Z*(*τ*) = *l*_*α*_(*τ*)/[*E*_0_($l_{\alpha }^{2}$(*τ*) | *Y*)]^1/2^.

Generation-specific effects in extended pedigrees were allowed in estimating the mean and variance of each trait, while the phenotypic correlations *ρ*_1 _for grandparent-grandchild and *ρ*_2 _for sibs were estimated by maximum-likelihood estimation (MLE) with the genetically natural constraint *ρ*_1 _≤ *ρ*_2_/2. The sex-average genetic map provided to Genetic Analysis Workshop 15 by Sung et al. [5] was used. The expected IBD counts were computed by MERLIN [6] using all 2819 SNP markers and full pedigrees. The score statistic was then computed at marker locations using the estimated IBD counts. Let *Z*_*n*_(*t*) be the score statistic at marker *t *for the *n*^th ^phenotype. We defined the genome scan statistic to be *Z*_*n *_= max_*t*_*Z*_*n*_(*t*) over all marker loci for trait *n*. The genome-wide *p*-value for each *Z*_*n *_(i.e., for each of the 3554 traits) was then computed using the skewness correction described in the Appendix.

Our theoretical calculation shows that, for these 14 pedigrees, using only sibships causes a loss of power equal to roughly 35% of the sample size. Here, we compare linkage scores for sibships and for entire pedigrees, and then we use the more powerful pedigree-based tests for analysis of the effects of our correction procedures.

### Control FDR based on the empirical null distribution

One useful method for addressing the multiple testing problem is to control the FDR [7]. For our problem, a successful FDR procedure requires 1) accurate evaluation of the genome-wide *p*-value for each trait, and 2) adjustment for the correlations among the genome scan test statistics. Here, we correct FDR using Efron's method to estimate the empirical null distribution [4]. For trait *n*, we computed the genome scan statistic *Z*_*n *_and approximated the genome-wide *p*-value *p*_*n *_using the skewness-correction method described in the Appendix (accuracy is checked using a Monte Carlo simulation). We then transformed *p*_*n *_to the normal quantile *q*_*n *_= Φ^-1^(1 - *p*_*n*_) and applied Efron's method on {*q*_*n*_} to estimate the empirical null distribution *N*(*μ*, *σ*^2^) The expected number of false positives for threshold *q *is 3554Φ((*q *- *μ*)/*σ*) and the FDR is estimated as FDR = 3554Φ((*q *- *μ*)/*σ*)/#{*q*_*n *_> *q*}. Here, we have implicitly assumed that the proportion of traits without linkage signals is close to one.

### Control FDR using permutations

To validate the FDR results obtained by correcting for skewness and for the empirical null distribution, we used 1000 permutations to determine the number of false positives and hence the FDR following Efron's method [3]. For permutation *n*, we computed the genome scan statistics $Z_{1}^{n},\cdots ,Z_{3554}^{n}$, and computed $Y_{0}^{n}=\#\{Z_{k}^{n}<3.5\}$ and $Y_{1}^{n}(b)=\#\{Z_{k}^{n}>b\}$ for *b *> 3.5, where 3.5 is the median value of *Z*. The correlation among the genome scan statistics causes $Y_{0}^{n},Y_{1}^{n}(b)$ to be correlated. So we can fit a linear regression model *Y*_1_(*b*) = *a*_1 _+ *a*_2_*Y*_0 _+ *ε *to the 1000 pairs of ($Y_{0}^{n},Y_{1}^{n}(b)$). For the observed data, we computed the genome scan statistics, *Y*_0 _and *Y*_1_, then computed the expected number of false positives among the *Y*_1_(*b*) positive findings as *a*_1 _+ *a*_2_*Y*_0_. The estimated FDR for threshold *b *was then (*a*_1 _+ *a*_2_*Y*_0_)/*Y*_1_. The permutation-based FDR procedure does not require accurate evaluation of genome-wide *p*-values or appropriate correction for the correlations among tests, but it is computationally intensive.

### Search for *cis*-regulated genes

If most linkages prove to be in *cis *regions, then the power to detect these linkages could be increased by considering only the markers within 5 Mb of that gene, because genome-wide *p*-values would have to be corrected only for this small proportion of markers for each expression trait. We searched the location of the 3554 gene names on . The markers within 5 Mb of each target gene were identified and the scan statistic was obtained as the maximum score for these markers. Because the number of markers and the genetic lengths in the *cis *region are highly variable, we evaluated the region-wide *p*-values empirically. We ran 8000 permutations and fit a quadratic curve (log *p*_*n *_= *α*_*n*,0 _+ *α*_*n*,1_*b *+ *α*_*n*,2_*b*^2^) to the results to predict the region-wide *p*-value for the *n*^th ^trait. The form of the *p*-value is suggested by the formula in Appendix. We then transformed *p*_*n *_to normal quantile *q*_*n *_and estimated the empirical null distribution based on the quantitles. The expected number of false positives and the FDR are based on the estimated empirical null distribution.

## Results

### Genome-wide *p*-value

To evaluate the validity of the skewness correction procedure described in the Appendix, we used that procedure to estimate the genome-wide *p*-value associated with the LOD score threshold of 5.3 (*Z *= 4.94) assumed by Morley et al. to have a genome-wide *p*-value of 0.001 based on Gaussian process theory [1]. Our skewness-correction procedure, however, determined that *Z *= 4.94 has a genome-wide *p*-value of 0.016. We then carried out 1600 Monte Carlo simulations (assuming no linkage) of genotypes for 2800 SNP markers (with minor allele frequencies from 0.25 to 0.5 assigned randomly) at 1.2-cM spacing, in 14 families with eight siblings and two parents per family. The genome-wide *p*-value for *Z *= 4.94 was found to be 0.0195 (SD = 0.0035), in close agreement with the skewness-corrected theoretical result, which supports the validity of the correction. Note that in the remainder of our analyses, we applied an FDR threshold of 10% (*Z *= 6.4) rather than a *p*-value threshold. When a large number of true positive results is expected, assessing significance by FDR might have more practical value than a family-wise error rate.

### Analysis of sibships vs. pedigrees

**Figure 1:**
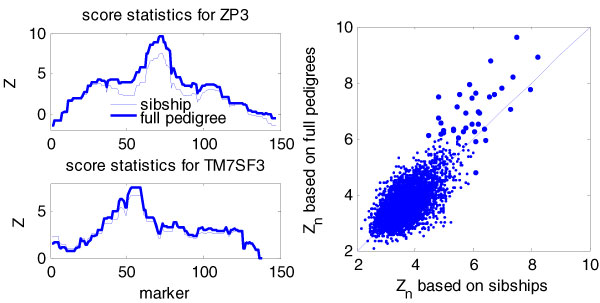
**Full pedigree analysis is more powerful than sibship analysis**. The left panel gives the test profiles for two traits reported in Morley et al. [1]. The right panel gives the scatter plot of the scan statistics using full pedigree and sibships. For largest values of the statistics (very likely to be true positives), most of the points are above the 45° line, which suggests that full pedigree analysis provides more power than sibship analysis to detect true linkages.

Figure 1 illustrates differences between linkage results for sibships and for pedigrees. In the two panels to the left, the score statistic results are shown for two expressed genes (*ZP3 *and *TM7SF3*) for the chromosome on which the gene is located, with the gene location in these two cases being directly under the peak score. Larger scores are observed for full pedigree analysis. In the panel to the right, the score based on sibship data is plotted against the score based on full pedigree data for 3554 traits. The largest (most significant) scores are larger using the full pedigree data.

### Corrected vs. uncorrected linkage results

**Figure 2:**
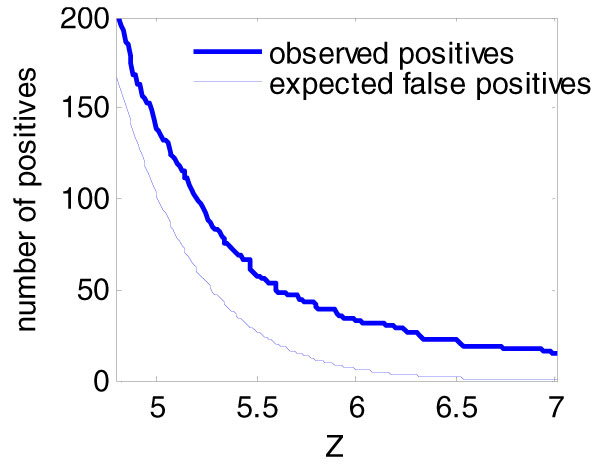
**False discovery rate threshold (analysis of full pedigrees)**. Shown are the numbers of expected false-positive findings and of observed positive findings, for each threshold of *Z *estimated using our corrected method (full pedigree analysis). The threshold for FDR = 10% is *Z *= 6.4. At this threshold we detected 22 significant linkages and expect that 2.2 are false positives. See text and Table 1 for comparison with uncorrected results.

Following Efron [4], we estimated the empirical null to be *N*(0.25, 1.05^2^). The *Z*_*n *_threshold of 6.4 for FDR = 10% was determined as shown in Figure 2. Using this threshold, we observed 22 gene expression levels with significant evidence of linkage (Tables 1 and 2). Among those genes, three were mapped to *trans *loci and 19 to *cis *loci. Using only sibships, we applied the same procedure and found evidence of linkage for only six genes at FDR = 10%. Similar results were obtained using permutation-based FDR method: 19 gene expression levels have significant evidence of linkage, among which 2 were mapped to *trans *loci and 17 to *cis *loci.

**Table 1:** Significant results in the uncorrected, corrected, and *cis*-only analyses

	Number of genes with a significant linkage signal				
	
Analysis	*cis*	*trans*	*cis *and *trans*	Multiple *trans*	Total
Uncorrected^a^	27	110	2	3	142
Corrected^b^	19^c^	3	0	0	22
Corrected (*cis *only)^d^	46	--	--	--	46

^a^Uncorrected analysis of Morley et al. [1]

^b^Corrected method described in this paper

^c^The 19 *cis *signals in the corrected analysis are a subset of the 27 in the uncorrected analysis, which are a subset of the 46 in the *cis*-only analysis.

^d^Corrected method when only the 10-Mb region around each gene is considered (and thus the correction for multiple tests is less severe).

**Table 2:** Expression phenotypes with significant linkage signals

Gene	Location	*Z*	*cis/trans*
*ZP3*	7q11.23	9.62	*cis*
*LRAP*	5q15	8.92	*cis*
*LOC388796*	20q11.23	8.79	*cis*
*HLA-DQB1*	6p21.3	8.18	*cis*
*RPL31*	2q11.2	7.92	*cis*
*HSD17B12*	11p11.2	7.82	*cis*
*CHI3L2*	1p13.3	7.78	*cis*
*EIF5A*	17p13	7.61	*cis*
*CSTB*	21q22.3	7.59	*cis*
*TM7SF3*	12q11	7.56	*cis*
*CGI-96*	22q13.2	7.50	*cis*
*HLA-DPB1*	6p21.3	7.47	*cis*
*DDX17*	22q13.1	7.42	*cis*
*EGR2*	10q21.1	7.15	*trans*
*DSCR2*	21q22.3	7.03	*trans*
*PEX6*	6p21.1	6.98	*cis*
*TGB1BP1*	2p25.2	6.92	*cis*
*PSPH*	7p15.2	6.90	*cis*
*PARP4*	13q11	6.72	*cis*
*AP3S2*	15q26.1	6.54	*cis*
*TGIF*	18p11.3	6.52	*trans*
*CPNE1*	20q11.22	6.51	*cis*

Shown are the 22 linkages detected by our corrected method (FDR = 10%). The first 19 of these were also detected using the permutation-based procedure (FDR = 10%).

Table 1 shows, for comparison, the results of the uncorrected analysis reported by Morley et al. [1]. Using a *Z*_*n *_threshold of 4.94 (LOD = 5.3 using regression statistics and an assumption of a Gaussian distribution), they reported 142 significant linkages, most of them *trans*, and calculated FDR = 2.5%. However, using the method described above, we expect 50 false-positive results at a threshold of 4.94 after correcting only for skewness, and 110 such results after further correcting for the empirical null. Therefore, we estimate FDR = 77.5% for the results reported by Morley et al. [1].

**Figure 3:**
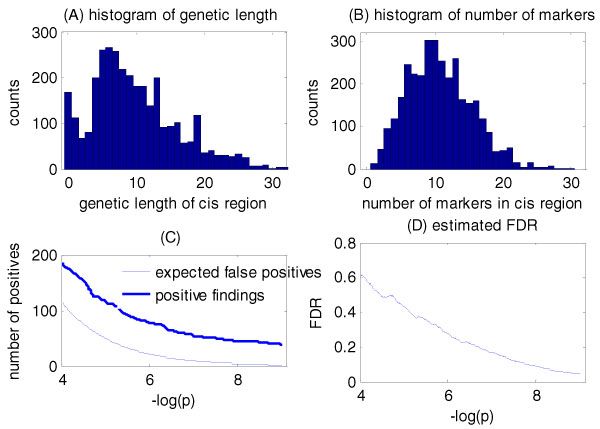
**Results of *cis*-only analysis**. Histogram of genetic lengths (A) and marker numbers (B) of 3554 *cis *regions. C, number of positive findings and expected false positives using our corrected method. D, Estimated FDR curve. We identified 46 significant *cis *linkages at threshold of region-wide *p *= 0.00036 or log(*p*) = -7.93 (FDR = 10%).

Finally, Figure 3 and Table 1 summarize the results of the corrected analysis limited to *cis *regions (within 5 Mb of each gene). Because this procedure maximizes *Z*_*n *_over a smaller number of tests, it detected 46 significant linkages at FDR = 10%.

## Discussion

We have addressed two issues relevant to linkage analysis of multiple traits. First, in data from family constellations larger than one sibling pair, the dependence of IBD sharing for different pairs of individuals within each family will create right-skewed score and regression tests, which we corrected using the method of Tang and Siegmund [2]. Second, when many tests are carried out, and there are correlations among tests, the distribution of test statistics under the null hypothesis can deviate in either direction from Gaussian expectation, which we corrected by the method of Efron [4]. We show that very similar results are obtained by applying these corrections to the data or by computing FDR empirically by permutation. This suggests that our corrections are valid and can be used in place of the very time-consuming permutation procedure.

Our analyses detected far fewer significant tests than the analysis of Morley et al. [1]. There are several differences between these analyses: they selected 142 linkage signals based on a genome-wide *p*-value threshold of 0.001, but without correcting for skewness; and they computed the expected number of false positives by multiplying this *p*-value by the number of tests, without correcting for the correlations among tests. Our results may be more plausible biologically, in that most of the significant linkages of expression levels are *cis*, i.e., close to the gene, where regulatory elements are known to exist. This is consistent with the result of a larger recent gene expression linkage study [8] of 20,413 transcripts in 1200 individuals from 40 Mexican-American families, where 95% of LOD scores >5.0 were located in the *cis *region of the expressed gene.

We would therefore suggest that in linkage studies of correlated traits in larger families, more accurate genome-wide inferences can be made if *p*-values are corrected for skewness caused by correlations of IBD sharing proportions for pairs of relatives, and if the expected proportion of false-positive results is corrected based on the empirical null distribution of test statistics. This proposal requires further testing where the "true" positives are known, using simulation of both expression levels and marker genotypes or using data for linkages that have been validated biologically.

## Competing interests

The author(s) declare that they have no competing interests.

## Appendix

Given skewness *γ*, inter-marker distance Δ, genetic length of the genome *L*, and the average recombination rate *β*, the genome-wide *p*-value can be approximated by

$$P\{\max_{t}Z(t)>b\}\approx 1-\exp\left(-[22(1-\Phi (-b))+L\beta \varphi (b)\nu (b\sqrt{2\beta \Delta })]\frac{\exp(\phi (\xi )-\xi b+b^{2}/2)}{\sqrt{1+\gamma \xi }}\right),$$

where *ϕ*(*ξ*) = *E*exp(*ξZ*(*t*)) ≈ *ξ*^2^/2 + *γξ*^3^/6 and *ξ *is chosen as the solution of *ϕ*'(*ξ*) = *ξ *+ *γξ*^2^/2 = *b*. The function $\nu (x)=2x^{-2}[-2\sum_{k=1}^{+\infty }\Phi (-xk^{1/2}/2)]$[9] for *x *> 0. It can be approximated by *ν*(*x*) ≈ exp(-0.583*x*) very accurately for 0 <*x *< 2; the series converge fast for large *x*. For GAW15 linkage data, *β *= 0.033, *γ *= 0.427 (detail omitted), and Δ = 3300 cM/2819 ≈ 1.2 cM.

## References

[B1] Morley M, Molony CM, Teresa M, Weber TM, Devlin JL, Ewens KG, Spielman RS, Cheung VG (2004). Genetic analysis of genome-wide variation in human gene expression. Nature.

[B2] Tang H-K, Siegmund D (2001). Mapping quantitative trait loci in oligogenic models. Biostatistics.

[B3] Efron B (2007). Correlation and large-scale simultaneous significance testing. J Am Stat Assoc.

[B4] Efron B (2004). Large-scale simultaneous hypothesis testing: the choice of a null hypothesis. J Am Stat Assoc.

[B5] Sung Y, Di Y, Fu AQ, Rothstein JH, Sieh W, Tong L, Thompson EA, Wijsman EM (2007). Comparison of multipoint linkage analyses for quantitative traits in the CEPH data: parametric LOD scores, variance components LOD scores, and Bayes factors. BMC Proc.

[B6] Abecasis GR, Cherny SS, Cookson WO, Cardon LR (2002). Merlin-rapid analysis of dense genetic maps using sparse gene flow trees. Nat Genet.

[B7] Benjamini Y, Hochberg Y (1995). Controlling the false discovery rate: a practical and powerful approach to multiple testing. J R Stat Soc Ser B.

[B8] Göring HHH, Curran JE, Johnson MP, Dyer TD, Jowett JBM, Mahaney MC, MacCluer JW, Collier GR, Moses EK, Blangero J (2006). Large-scale genetic investigation of genome-wide transcriptional profiles [abstract]. Annual Meeting of The American Society of Human Genetics, 10–14 October 2006; New Orleans.

[B9] Siegmund D (1985). Sequential Analysis: Tests and Confidence Intervals.

